# Assessment of a novel bile solubility test and MALDI-TOF for the differentiation of *Streptococcus pneumoniae* from other mitis group streptococci

**DOI:** 10.1038/s41598-017-07772-x

**Published:** 2017-08-02

**Authors:** Hans-Christian Slotved, Richard R. Facklam, Kurt Fuursted

**Affiliations:** 1 Department of Bacteria, Parasites and Fungi, Statens Serum Institut, Copenhagen, Denmark; 2 Retired, Centers for Disease Control and Prevention, Atlanta, GA USA

## Abstract

This study assesses a novel bile solubility test and MALDI-TOF for the differentiation of *Streptococcus pneumoniae* from other mitis group streptococci, including differentiation of *S. pneumoniae* from *Streptococcus pseudopneumoniae*. Eighty-four species verified mitis group isolates were subjected to our bile solubility test (which measures and calculates the differences of absorbance in the test tube containing 10% sodium deoxycholate versus a blank control tube, after incubation for 10 minutes at 36 °C using a spectrophotometer) and MALDI-TOF MS (both the standard result output and by visual spectra evaluation). Applying a calculated optimal cut-off absorbance-value of 2.1, differentiated *S. pneumoniae* from all but one other mitis group streptococci (one *S. mitis* isolate generated an OD-value above 2.1). MALDI-TOF score value identification identified correctly 46 *S. pneumoniae* and 4 *S. pseudopneumoniae* but misidentified 16 other mitis group strains. Visual spectra evaluation correctly identified all *S. pneumoniae* and *S. pseudopneumoniae* strains but misidentified 13 other mitis group strains. The bile solubility test based on spectrophotometric reading described in this study can differentiate *S. pneumoniae* from other *Streptococcus* species. Combining the bile solubility test and the MALDI-TOF spectra results provide a correct identification of all *S. pneumoniae* and *S. pseudopneumoniae* isolates.

## Introduction

Worldwide, *Streptococcus pneumoniae* (pneumococci) infections cause high morbidity and mortality among children and elderly^1^. Invasive pneumococcal disease (IPD) is one of the most frequent types of bacteraemia and meningitis in infants globally as well as in Denmark^2, 3^. It is therefore essential to be able to identify *S. pneumoniae* easily and rapidly^4, 5^.

The detection and differentiation of the mitis group and in particular between the two species *S. pneumoniae* and *Streptococcus pseudopneumoniae* can be a very difficult task and often requires several different methods^4, 5^. Before reaching the point of pneumococcal serotyping identification, it is important to correctly identify *S. pneumoniae* from other mitis group streptococci. In general, methods such as the bile solubility test, optochin susceptibility, and colony morphology have been the methods of choice for species differentiation^5, 6^. In recent years, other methods, such as MALDI-TOF MS (matrix-assisted laser desorption/ionization time-of-flight mass spectrometry) identification based on spectra and molecular methods have been described and used for streptococcal species identification^7, 8^. However these methods have also been shown to have problems with false identification results^9–11^.

The bile solubility test is generally considered to be an accurate test for differentiating *S. pneumoniae* from other mitis group streptococci, including *S. pseudopneumoniae* ^4, 5^. The test results from the method is however difficult to interpret, particularly because it is based on a subjective human evaluation^5^. While the MALDI-TOF procedure is considered a more simple method of identification and is used in many clinical laboratories, correct identification of mitis group streptococci (*S. pneumoniae* and *S. pseudopneumoniae* in particular) is still a difficult task^11^.

In this study we describe a bile solubility test, which is not based on subjective human evaluation. The described bile solubility test uses a densitometer and establishes a standardized bacterial concentration and provides an optical density value (OD-value) for the bile solubility test. Furthermore, this study presents data establishing a cut-off OD-value which can differentiate *S. pneumoniae* from other mitis group streptococci, including *S. pseudopneumoniae*. We also evaluate species identification based on both MALDI-TOF score values and MALDI-TOF visual spectra evaluation. Both methods are tested against species verified isolates belonging to the mitis group. Finally we present a recommendation of how to identify/differentiate species within the mitis group in a clinical setting.

## Materials and Methods

### Clinical isolates

A total of 84 mitis group strains were tested, of which 47 were *S. pneumoniae* strains (22 serotype positive and 25 confirmed nontypeable pneumococci), nine strains were *S. pseudopneumoniae*, 20 strains were *Streptococcus mitis*, two strains were *Streptococcus oralis*, four strains were *Streptococcus sanguinis*, and two strains were *Streptococcus australis*.

Twenty-four strains including the species *S. pneumoniae*, *S. mitis* and *S. pseudopneumoniae* were obtained from the strain collection described by Kilian *et al*.^12^.

Fifteen non-capsular pneumococcal strains were obtained from The Centers for Disease Control and Prevention, Atlanta GA, USA. The isolates were identified using the PCR procedure described by Park *et al*.^13^.

Thirty strains including the species *S. pneumoniae*, *S. pseudopneumoniae*, *S. mitis*, *S. oralis*, *S. sanguinis*, and *S. australis* were obtained from the NSR laboratory, Statens Serum Institut (SSI). The isolates were identified using multilocus sequence analysis (MLSA)^14^ and the 16*S* rRNA-based molecular procedures^12, 15^.

Thirteen strains were capsular strains (Sanger strains) described by Bentley *et al*.^16^, representing the serotypes included in the PCV 13.

Two reference strains, *S. pneumoniae* ATCC49619 and *S. pseudopneumoniae* CCUG49455, were also included.

Three *S. mitis* strains were confirmed to have the *lytA* gene by molecular sequence procedures described in previous studies^12, 15^, two strains were from the strain collection described by Kilian *et al*.^12^ and one strain was obtained from the NSR laboratory, Statens Serum Institut.

### The bile solubility testing

An inoculum was prepared from colonies obtained from overnight incubated blood agar plates (36 °C) in a tube with 2 ml of saline adjusting the cell density to a density of McFarland 4 using a spectrophotometer (Biomerieux, Densimat, Italy). The suspension was then divided equally into two tubes each containing each 1 ml. 200 µl of 10% sodium deoxycholate was added to the test tube and 200 µl of saline was added to the control tube. Both tubes were incubated for 10 minutes at 36 °C (stationary), before the absorbance of both were measured spectrophotometrically. The difference in absorbance of the test tube versus the control tube was calculated as an OD-value. A negative difference between the test tube and the blank control was set to 0.0 OD-value.

Twenty-five isolates consisting of *S. pneumoniae* (11 strains), *S. pseudopneumoniae* (2 strains) and *S. mitis* (12 strains) were tested four times on average, by three different people, over a six-month period.

### MALDI-TOF MS identification

MALDI-TOF MS (matrix-assisted laser desorption/ionization time-of-flight mass spectrometry) (Bruker Daltonics; Compass 1.4, Version 3.4, Build 3.4.76.0) was performed on strains transferred directly from bacterial colonies. Species identification by MALDI-TOF MS was based either on the standard MALDI-TOF score value (Biotyper database version MBT 6903 MSP Library (#1829023)) or on a visual inspection for the presence/absence of peak pairs of *m/z* 2625, 2911, 2937.5, 5253, 5824, 5877 and 6955 as described by Werno *et al*.^7^.

### Data analysis

Data were analysed using GraphPad Prism version 5 (GraphPad Software) for descriptive statistical analysis. All calculations of median and confidence interval (CI) were performed using the R version 3.2.4 for Windows (http://www.r-project.org/). All negative calculated OD-values were set to 0.0 OD. The Wilcoxon Rank-Sum (Mann-Whitney U) test in R was used to calculate P-values with C.I. P < 0.05 was considered significant. The Receiver Operating Characteristic (ROC) in R (pROC)^17^ was used to calculate the ROC values and C.I. values. For the optimal cut-off values were employed “closest.topleft” defined as the optimal threshold point closest to the top-left of the graph showing perfect sensitivity (X-axis) or specificity (Y-axis)^17^.

## Results

### Supplementary Tables 1 and 2 present specific and detailed bile and MALDI-TOF data for each tested strain

Depicted in Table 1 are the OD-values obtained from the bile solubility test when applied to the strains included in the study.

**Table 1:** Mean OD-values for the species tested in the study. The species annotation of strains was based on MALDI-TOF output and spectra. For detailed results per species, see supplementary Table 1.

Strain ID	Number of strains	OD Bile Solubility test	Median OD-value with CI
		Range	
*S. pneumoniae*, typeable	22	2.6–3.4	3.0 (95% CI: 2.9–3.1)
*S. pneumoniae*, nontypeable	25	2.3–3.3	2.9 (95% CI: 2.8–3.1)
*S. pneumoniae*, all isolates	47	2.3–3.4	3.0 (95% CI: 2.9–3.1)
*S. pseudopneumoniae*	9	0.3–2.0	1.8 (95% CI: 1.0 – 1.8)
^1^ *S. mitis*	20	0–2.55	0.1 (95% CI: 0.1–0.3)
^2^Other *Streptococcus* species	8	0 – 0.3	0.1 (95% CI: 0.00–0.3)

^1^Three strains were autolysin *(lytA)* positive.

^2^Four *S. sanguinis*, two *S*. *australis*, two *S*. *oralis*.

The *S. pneumoniae* bile test showed a median OD-value of 3.0 (95% CI: 2.9–3.1) within the range of 2.6–3.4 with no significant difference (P = 0.28 95% CI: −0.1–0.25) between typeable *S. pneumoniae* (median OD value = 3.0 (95% CI: 2.9–3.1)) and non-serotypeable *S. pneumoniae* (median OD value = 2.9 (95% CI: 2.8–3.1)) (Table 1).

*S. pseudopneumoniae* strains showed a median OD-value 1.8 (95% CI: 1.0–1.8) within the range of 0.3–2.0 (Table 1).

*S. mitis* showed a median OD-value of 0.1 (95% CI: 0.1–0.3) within the range of 0–2.55. Of the 20 tested *S. mitis* strains, three isolates showed a high OD-value (above 1.0 OD). These three isolates were all found to contain the autolysin gene. The remaining *Streptococcus* species (*S. oralis*, *S. sanguinis*, *S. australis*) showed a median OD-value of 0.1 (95% CI: 0.0–0.3) within the range of 0.0–0.3 (Table 1).

Species identification based on MALDI-TOF score values was performed on all strains (Table 2). All pneumococcal isolates with a serotypeable capsule were correctly identified, while one non-capsular isolate was incorrectly identified as *S. pseudopneumoniae* (Table 2). Based on the score value, four of the nine *S. pseudopneumoniae* strains were correctly identified as *S. pseudopneumoniae*, while the remaining five were incorrectly identified as either *S. oralis* or *S. pneumoniae* (Table 2). Since we have not evaluated the standard MALDI-TOF database output regarding the remaining species tested in the study, correct species identification cannot be expected, but instead provides suggested annotations which need further confirmation. Of the 20* S. mitis* strains, the score value correctly identified 10 of the 20 tested isolates, while the rest were scored as six *S. oralis*, one *S. pseudopneumoniae* and three *S. pneumoniae*. All *S. oralis* and *S. sanguinis* strains were correctly identified by the MALDI-TOF score value.

**Table 2:** Comparison of species identification by official (Score value) and visual interpretations of MALDI-TOF seven profile peaks, with species annotation based on the m/z values described by Werno *et al*.^7^.

Confirmed ID	m/z value							Test results based on score value	Test results based on visual evaluation value
Profile	2625	2911	2937.5	5253	5824	5877	6955	Score tested/correct	Visual tested/correct
*S. pneumoniae (typeable)*	−	−	+	−	−	+	−	22/22	22/22
*S. pneumoniae* (nontypeable)	−	−	+	−	−	+	−	25/24^1^	25/25
*S. pseudopneumoniae*	+	−	+	+	−	+/−	−	9/4^2^	9/9
*S. mitis*	+ /−	+/−	+/−	+/−	+/−	+/−	+/−	20/10^3^	20/14^4^
*S. oralis*	−	+	−	−	+	−	+/−	2/2	2/1^5^
*S. australis*	−	−	−	−	−	−	−	2/0^6^	2/0^7^
*S. sanguinis*	−	−	−	+/−	−	−	+/−	4/4	4/0^8^

+: only strains with peaks were found.+/−: strains both with and without peaks were detected. −: no strains with peaks were detected.

^1^One specimen identified as *S. pseudopneumoniae*.

^2^Four isolates identified as *S. pneumoniae*, one ID as *S. oralis*.

^3^Six isolates identified as *S. oralis*, three isolate ID as *S. pneumoniae*, one isolate ID as *S. pseudopneumoniae*.

^4^Three isolates identified as *S. pneumoniae*, three *S. mitis*/*S. oralis*.

^5^One isolate identified as *S. mitis*/*S. oralis*.

^6^Both isolates identified as *S. parasanguinis*.

^7^Both isolates could not be identified.

^8^All isolates could not be identified.

The species identification based on the MALDI-TOF score value only showed correct species identification if the top ten listed species with a score value above 1 was the same species. If more species were recommended with a score value above 1, misidentification was observed (Table 2, Supplementary Table 1).

Evaluating strain identification based on visual inspection of the seven peaks from the MALDI-TOF spectra^7^ showed a more precise identification of some of the tested isolates (Table 2). All pneumococcal isolates, both capsular and non-capsular isolates, showed peaks at *m/z* 2937.5 and *m/z* 5877 respectively, which are the characteristic peaks for pneumococcal isolates. Of the remaining streptococcal isolates three *S. mitis* isolates showed only peaks at *m/z* 2937.5 and *m/z* 5877 and could therefore not be differentiated from the *S. pneumoniae* isolates. All nine *S. pseudopneumoniae* were correctly identified based on the spectra, where eight of the nine isolates showed a peak at *m/z* 2625, 2937.5, 5253 and 5877, while one isolate did not show a peak at *m/z* 5877. This peak combination is described as characteristic for *S. pseudopneumoniae* and was not observed for any other tested isolates (Table 2, Supplementary Table 1).

By combining the bile OD-values and the MALDI-TOF results based on the evaluation of the seven peaks (Table 1, Table 2, Supplementary Table 1), we were able to identify correctly all *S. pneumoniae* isolates, if the bile OD-value was 2.1 or above and the isolate showed a peak at *m/z* 2937.5 and *m/z* 5877. All *S. pseudopneumoniae* were also correctly identified if the bile OD-value was between 0.9 and 2.1 and the isolate showed peaks at *m/z* 2625, 2937.5 and 5253.

The remaining tested strains belonging to the mitis group could not be differentiated into species based on evaluating the MALDI-TOF peaks and the bile OD-values without false negative identifications.

**Figure 1:**
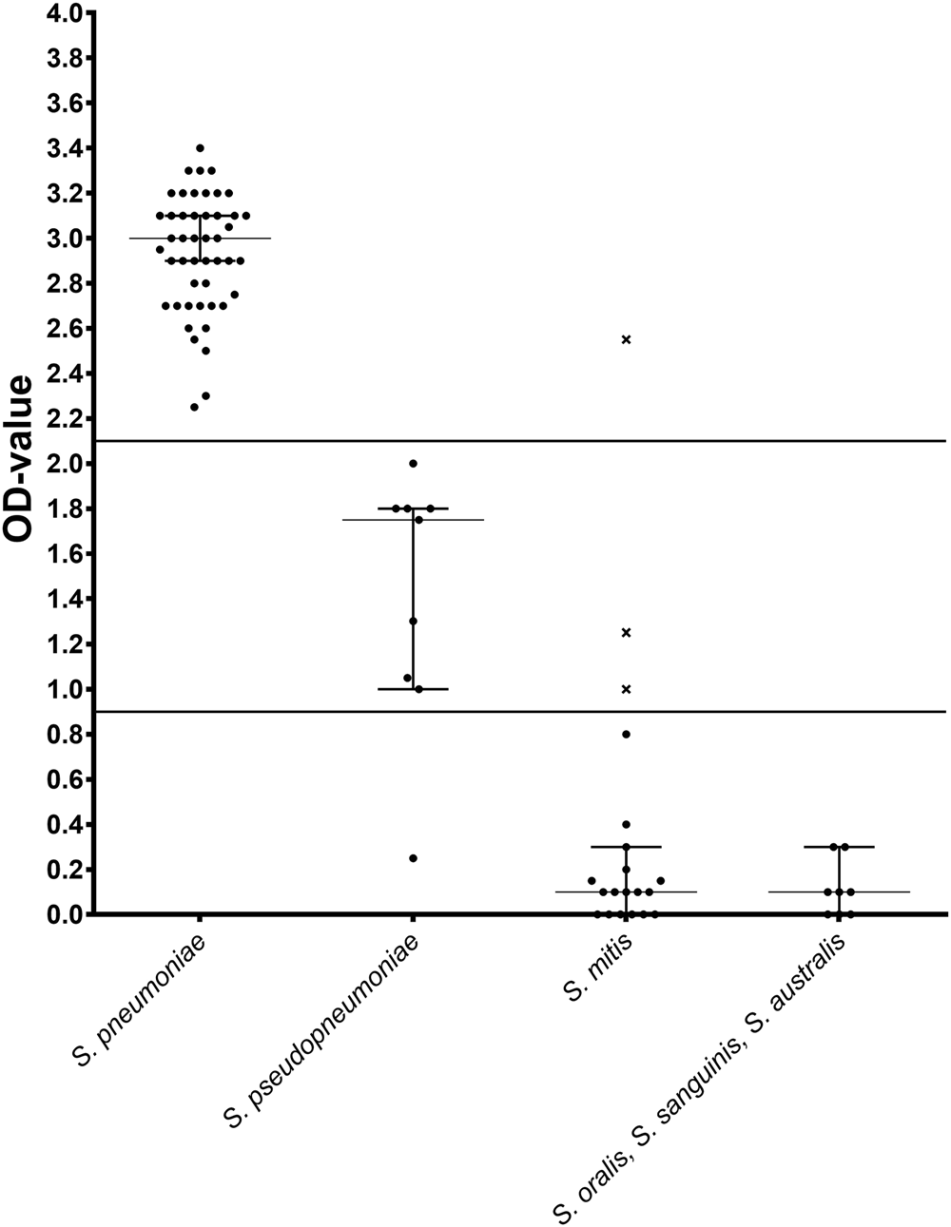
The median OD-value (95% CI) for six *Streptococcus* species. Lines indicate the calculated cut-off OD-values of 2.1 and 0.9. The three *S. mitis* strains containing the autolysin (*lytA*) gene are indicated by an ‘x’.

Table 3 and Fig. 1 present the optimal cut-off value and the Area Under the Curve (AUC) for differentiating all the tested strains, based on their bile solubility OD-values. Choosing a cut-off OD-value of 2.1 showed an excellent AUC of 100% (95% CI 100–100), which showed no false negative OD-values for nontypeable *S. pneumoniae*, while one *S. mitis* strain (OD-value of 2.7) showed OD-values above a cut-off value of 2.1 (Fig. 1). The median bile OD-value for nontypeable *S. pneumoniae* (median OD value = 2.9) differed significantly from the median bile OD-value for *S. pseudopneumoniae* (OD value = 1.8) (P = 0.0005, 95% CI 0.8–1.7).

**Table 3:** Area under the curve (AUC) and the optimal cut-off values calculated by using “closest topleft”.

Group 1 (n)	Group 2 (n)	% AUC (95% CI)	Differences of OD-values from the two groups	Optimal cut-off value
			P value (95% CI)	“closest.topleft”
*S. pneumoniae* nontypeable (25)	*S. pseudopneumoniae* (Highest values) (9)	100 (100 – 100)	P = 0.00001 (1.1–1.8)	2.13 (100.0% specificity. 100% sensitivity).
*S. pseudopneumoniae* (9)	*S. mitis* (20)	90.83 (79.38 – 100)	P = 0.0005 (0.8–1.7)	0.9 (66.67% specificity. 65.0% sensitivity).
*S. pseudopneumoniae* (9)	*S. mitis* (20). *S. sanguinis* (4). *S. australis* (2). *S. oralis* (2)	92.66 (83.6 – 100)	P = 0.0001 (0.9–1.7)	0.9 (66.67% specificity. 78.57% sensitivity).

Choosing a cut-off OD-value of 0.9 for differentiating *S. pseudopneumoniae* from *S. mitis*, showed a good AUC of 90.83% (95% CI 79.38–100). The median OD-value for *S. pseudopneumoniae* (median OD value = 1.8) differed significantly from the median bile OD-value for *S. mitis* (OD value = 0.1) (P = 0.0005, 95% CI 0.8–1.7).

Choosing a cut-off OD-value of 0.9 for differentiating *S. pseudopneumoniae* from the other streptococcal species tested (*S. oralis*, S*. sanguinis*, *S. australis)* showed a good AUC of 92.66% (95% CI 83.6–100). The median bile OD-value for *S. pseudopneumoniae* (OD value = 1.8) differed significantly from the median bile OD-value for other strains (*S. oralis*, S*. sanguinis*, *S. australis)* (OD value = 0.1) (P = 0.0001, 95% CI 0.9–1.7).

Comparing the median bile OD-value for *S. mitis* (OD value = 0.1) with the combined median bile OD-value for other strains (*S. oralis*, S*. sanguinis*, *S. australis)* (OD value = 0.1) showed no significant difference, P = 0.4 and a CI of (95%, CI −0.1–0.2). The AUC was not calculated.

Table 4 and Supplementary Table 2 summarise the reproducibility of the data from repeated OD values of 25 strains of *S. pneumoniae*, *S. pseudopneumoniae*, and *S. mitis*. The number of tests is limited and therefore a statistical test has not been performed on the results. The isolates were tested for their bile OD-value repeatedly over approximately 6 months by different persons. It was found that the variation of the OD values in general for pneumococci was within the range of 0.6, although one strain showed a range of 1.1. The percentage of coefficient of variation (CV) for the tested *S. pneumoniae* isolates showed low variation, with the highest % CV found to be 17.74. Two *S. pseudopneumoniae* strains showed a range of 0.4. The highest % CV for the two strains was 75.9.

**Table 4:** Repeated testing of 25 strains over time and performed by different persons. OD-values below 0 are considered as 0.0. For detailed data see supplementary Table 2.

		Number of tests	Total range of test (OD-values)	Median OD-value	% CV
*S. pneumoniae*	ATCC49619	7	2.9–3.5 (0.6)	3.3	6.86
*S. pneumoniae* Non-capsular	Kilian/13725	4	2.2–3.3 (1.1)	2.85	17.74
*S. pneumoniae* Non-capsular	Kilian/14860	4	2.9–3.3 (0.4)	3.1	5.89
*S. pneumoniae* Non-capsular	Kilian/A39557	4	2.1–2.5 (0.4)	2.3	7.938
*S. pneumoniae* Non-capsular	Kilian/A8061	4	2.4–2.8 (0.4)	2.6	6.28
*S. pneumoniae* Non-capsular	Kilian/A12931	4	3–3.6 (0.6)	3.15	8.16
*S. pneumoniae* Non-capsular	Kilian/A9003	4	2.9–3.2 (0.3)	3.0	4.16
*S. pneumoniae* Non-capsular	Kilian/A7890	4	2.8–3.1 (0.3)	2.9	4.30
*S. pneumoniae* Non-capsular	Kilian/A2009	4	2–2.5 (0.5)	2.2	9.97
*S. pneumoniae* Non-capsular	Kilian/A4708	4	2.8–3.4 (0.6)	3.05	8.96
*S. pneumoniae* Non-capsular	Kilian/A39363	4	2.4–2.9 (0.5)	2.75	8.00
*S. pseudopneumoniae*	Kilian/SK1516	4	1.2–1.4 (0.2)	1.4	7.41
*S. pseudopneumoniae*	Kilian/SK674	4	0 – 0.4 (0.4)	0.25	75.90
*S. mitis*	Kilian/SK642	4	0–0 (0)	0	0
*S. mitis*	Kilian/SK637	4	0–1.2 (1.2)	0.1	164.13
*S. mitis*	Kilian/SK271	4	0 – 0.2 (0.2)	0.05	127.66
*S. mitis*	Kilian/SK142	4	0–0.2 (0.2)	0	200.0
*S. mitis* (Autolysin gene)	Kilian/SK564	4	0.4-1.3 (0.9)	1	48.65
*S. mitis*	Kilian/SK137	4	0–0.2 (0.2)	0	200.0
*S. mitis*	Kilian/SK1126	4	0–0.2 (0.2)	0	200.0
*S. mitis* (Autolysin gene)	Kilian/SK597	4	0.2–1.4 (1.2)	0.7	78.88
*S. mitis*	Kilian/SK608	4	0–0.3 (0.3)	0.05	141.42
*S. mitis*	Kilian/SK321	4	0–0 (0)	0	0
*S. mitis*	Kilian/SK113	4	0–0.3 (0.3)	0.05	141.4
*S. mitis*	Kilian/SK578	2	0–0 (0)	0	0

The remaining strains tested showed large differentiations in the tested OD-values.

## Discussion

Since *S. pneumoniae* can cause severe human infections^18^, rapid and reliable species identification is essential^5, 7, 11^. However, differentiating *S. pneumoniae* from other mitis group streptococci (in particular the relatively newly described *S. pseudopneumoniae* ^19^) can be a very difficult task and often requires several different methods^4, 5^.

Many studies show that identification of *S. pneumoniae* based on only a single test method will give a bias of either false positive or false negative identifications and thereby a possible overestimation of a species^5, 9, 20^. Therefore, it is generally recommended to use several different methods for species identification^9, 20^, with the WHO recommended culture based pneumococcal identification in combination with both the optochin susceptibility and bile solubility test^6^. Conversely, Yahiaoui *et al*. recently recommended a new identification protocol, excluding the optochin susceptibility test, and instead recommending only the use of the bile solubility test for species identification^21^.

In this study we have presented a new objective and optimized the procedure for the bile solubility test, because it is generally considered to be one of the main standard tests for the identification of *S. pneumoniae* ^5, 6^. The advantages of using the bile solubility test is that it is a simple and reliable test^4^, which can be used in low tech laboratory facilities. The disadvantage of the bile solubility test generally used is that the test results are based on a visual interpretation, and the results are therefore imperilled to human subjective evaluation^5^. By using a densimat to measure both the amount of bacteria used for the bile solubility test and the final OD-value of the performed test, we removed the need for human evaluation and provide results solely based on instrumental measurement. Because the presented bile solubility procedure is based on instrumental measurement and not human evaluation, it was important to evaluate the test against strains verified for their identification. We did not test our bile procedure with the bile test based on visual evaluation, as the test results from a visually based bile test is very difficult to reproduce from one laboratory to another. The bile solubility test data presented in this study show that it is possible to obtain information (species identification as *S. pneumoniae*/*S. pseudopneumoniae*/other mitis group streptococci) from the OD-values of streptococcal species. Finding an OD-value of 2.1 or above will indicate that the species is correctly identified as *S. pneumoniae*, while finding an isolate with an OD-value in the range of 0.9 to 2.1 (Table 3) will suggest that it is an *S. pseudopneumoniae*. An OD-value below 0.9 will suggest that it is an isolate belonging to either *S. mitis*, *S. oralis*, S*. sanguinis*, or *S. australis*. A small study (Table 4) furthermore showed that the test was quite robust with only minor time and person variations on the OD-value.

The *lytA* gene (encoding autolysin) is said to be characteristic of *S. pneumoniae* and an indication of bile solubility; however, the presence of *lytA* has also been reported in other mitis group streptococci including *S. pseudopneumoniae* and *S. mitis* ^9, 19^. In this study we found that the three *S. mitis* isolates showing an OD-value within the range of 1.3 to 2.7 all contained the gene for autolysin (Fig. 1, Supplementary Table 1). Although the data are currently very limited, an unusually high OD-value observed for an *S. mitis* strain could possibly provide a marker for the presence of the *lytA* gene.

We also tested all the characterized isolates included in this study using MALDI-TOF. We found that species identification based on MALDI-TOF score value could only be recommended if the top ten species with a score value above 1 were the same species. If more species were recommended with a score value above 1, misidentification was observed. Further improvement of the MALDI-TOF database will improve the species identification and reduce misidentification including those observed in this study^11, 22, 23^. Instead of using the MALDI-TOF score value and including a visual evaluation of the MALDI-TOF spectra, we found that it was possible to identify both *S. pneumoniae* and *S. pseudopneumoniae* correctly by evaluating the seven peaks described by Werno *et al*.^7^. We found that three isolates of *S. mitis* were identified as *S. pneumoniae*. These three *S. mitis* strains showed identical peaks, characteristic for *S. pneumoniae* (Supplementary Table 1). We did not see any false positive identification of *S. pseudopneumoniae*. Using spectres can therefore be recommended for the identification of particularly *S. pseudopneumoniae* and *S. pneumoniae* strains; however, species identification based on the seven peaks is limited as it cannot be used to identify species such as *S. sanguinis*, where the MALDI-TOF score values correctly identified all four isolates.

**Figure 2:**
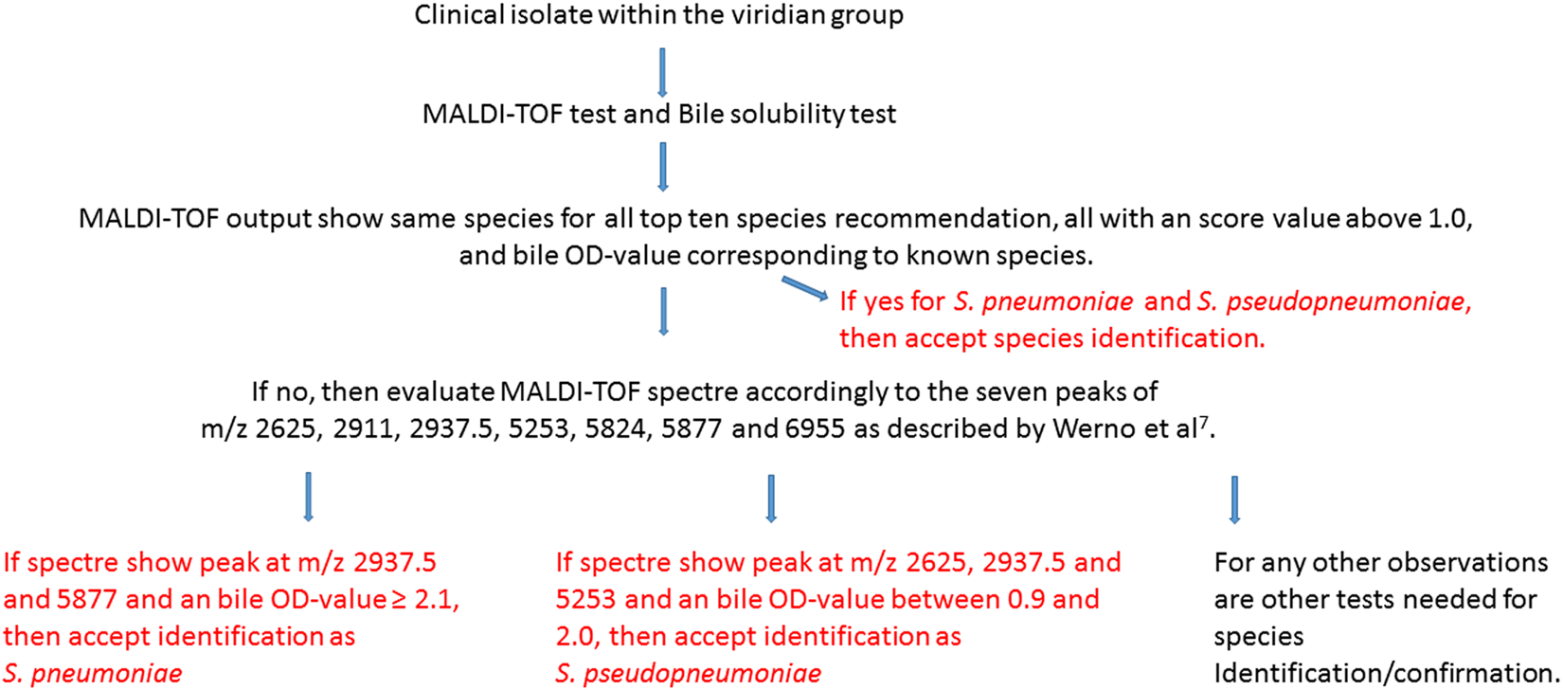
A description of species identification based on MALDI-TOF and Bile solubility test for clinical isolates from the mitis group.

Based on the results of this study we recommend a study workflow, utilising both the MALDI-TOF and bile solubility tests as presented in Fig. 2 as a means to test clinical mitis group isolates for correct annotation of *S. pneumoniae* and *S. pseudopneumoniae* strains. However, it has to be emphasised that the Fig. 2 flowchart is only based on the isolates tested in this study.

Species identification of isolates within the mitis group using the flowchart (Fig. 2) will be within the capacity of many clinical laboratories. For many clinical laboratories is it not a possibility to use molecular methods with a high enough specificity to correctly identify mitis group species due to time and expenses^11^. However it has to be emphasized that if the requirement described in the flowchart cannot be achieved for an isolate, other tests are needed for species identification^6^. As additional information, it has recently been recommended that the optochin test used by many diagnostic laboratories today as a part of routine identification of *S. pneumoniae*, is not used for the identification of *S. pneumoniae* ^21^. The presented methods can therefore be seen as alternatives to the optochin test.

Finally, it has to be stated by the authors that to obtain a fully correct identification of a streptococcal species within the mitis group, it is recommended to perform a molecular identification as described by Scholz *et al*.^15^. This identification procedure using NGS is, however, not within the capacity of many clinical settings.

It is advised that before using the described bile test procedure, it is recommended to evaluate the bile test against your own verified mitis group strains and the bile test procedure performed in the laboratory.

In conclusion this study presents data for a new objective bile solubility test based on instrumental measurement and not human interpretation. We present cut-off OD-values that can be used for differentiating/discriminating *S. pneumoniae* and *S. pseudopneumoniae* from other mitis group streptococci. Moreover, we confirmed the valuable use of visual MALDI-TOF spectra evaluation (as presented by Werno *et al*.^7^) for the identification of *S. pneumoniae* and *S. pseudopneumoniae*. We found that a combination of the identification data from these two tests provided a high rate of accurate identification of *S. pneumoniae* and *S. pseudopneumoniae*. We have presented a protocol (Fig. 2) using the combination of the MALDI-TOF test and the bile solubility test, which is very well suited for routine identification in clinical microbiology laboratory settings due to its quick and simple procedure.

## Electronic supplementary material

Supplementary table 1, Supplementary table 2
