# Supplementary material for: Assessment of a novel bile solubility test and MALDI-TOF for the differentiation of Streptococcus pneumoniae from other mitis group streptococci
Source: Sci Rep. 2017 Aug 2;7:7167. doi: 10.1038/s41598-017-07772-x (PMC5540920; doi:10.1038/s41598-017-07772-x)
Supplement: Supplementary file 1 — Supplementary table 1, Supplementary table 2 [file 41598_2017_7772_MOESM1_ESM.doc]

Assessment of a novel bile solubility test and MALDI-TOF for the differentiation of *Streptococcus pneumoniae* from other mitis group streptococci.

Hans-Christian Slotved^1^*, Richard R Facklam^2^, Kurt Fuursted^1^

^1^Department of Bacteria, Parasites and Fungi, Statens Serum Institut, Copenhagen, Denmark.

^2^Retired, Center for disease Control and Prevention, Atlanta, USA

Running title: bile solubility test

***Corresponding author:**

Hans-Christian Slotved

Department of Bacteria, Parasites and Fungi

Statens Serum Institut

Artillerivej 5, DK-2300 Copenhagen

Denmark

Phone: +45 32688422, Fax: +45 32683865, E-mail: hcs@ssi.dk.

**Supplementary tables**

Supplementary table 1. MALDI profiles both based on the MALTI-TOF Rank Quality system and visual evaluation on the peaks described by Werno et al^7^.

| *Defined species* | Strains identification | MALDI-TOF | Number of species with score value ≥ X  *Including correct species | | m/z value | | | | | | | MALDI-TOF species definition base on m/z value´s described by  Werno et al^7^ | Bile test range |
| --- | --- | --- | --- | --- | --- | --- | --- | --- | --- | --- | --- | --- | --- |
|  |  | (Top score) | ≥ 2.0 | ≥ 1.0 | 2625 | 2911 | 2937.5 | 5253 | 5824 | 5877 | 6955 |  |  |
| Serotype 19F | ATCC49619 | *S. pneumoniae* | 1* | - | - | - | + | - | - | + | - | *S. pneumoniae* | 2.9 – 3.5 |
| Serotype 24F | SSI/404-14 | *S. pneumoniae* | 1* | - | - | - | + | - | - | + | - | *S. pneumoniae* | 3.1 |
| Serotype 24F | SSI/669-14 | *S. pneumoniae* | 1* | - | - | - | + | - | - | + | - | *S. pneumoniae* | 2.9 |
| Serotype 24F | SSI/747-14 | *S. pneumoniae* | 1* | - | - | - | + | - | - | + | - | *S. pneumoniae* | 3.4 |
| Serotype 24F | SSI/90-15 | *S. pneumoniae* | 1* | 1* | - | - | + | - | - | + | - | *S. pneumoniae* | 3.1 |
| Serotype 24F | SSI/96-15 | *S. pneumoniae* | 1* | - | - | - | + | - | - | + | - | *S. pneumoniae* | 3.1 |
| Serotype 24F | SSI/182-15 | *S. pneumoniae* | 1* | 1* | - | - | + | - | - | + | - | *S. pneumoniae* | 3.1 |
| Serotype 24F | SSI/233-15 | *S. pneumoniae* | - | 1* | - | - | + | - | - | + | - | *S. pneumoniae* | 3 |
| Serotype 24F | SSI/641-15 | *S. pneumoniae* | 1* | 1* | - | - | + | - | - | + | - | *S. pneumoniae* | 3.3 |
| Serotype 1 | Sanger serotype 1 | *S. pneumoniae* | 1* | 1* | - | - | + | - | - | + | - | *S. pneumoniae* | 2.6 |
| Serotype 3 | Sanger serotype 3 | *S. pneumoniae* | - | 1* | - | - | + | - | - | + | - | *S. pneumoniae* | 2.9 |
| Serotype 4 | Sanger serotype 4 | *S. pneumoniae* | - | 2* | - | - | + | - | - | + | - | *S. pneumoniae* | 3.0 |
| Serotype 5 | Sanger serotype 5 | *S. pneumoniae* | 1* | - | - | - | + | - | - | + | - | *S. pneumoniae* | 3.1 |
| Serotype 6A | Sanger serotype 6A | *S. pneumoniae* | 1* | 1* | - | - | + | - | - | + | - | *S. pneumoniae* | 2.7 |
| Serotype 6B | Sanger serotype 6B | *S. pneumoniae* | - | 1* | - | - | + | - | - | + | - | *S. pneumoniae* | 2.7 |
| Serotype 7F | Sanger serotype 7F | *S. pneumoniae* | - | 2* | - | - | + | - | - | + | - | *S. pneumoniae* | 3.0 |
| Serotype 9V | Sanger serotype 9V | *S. pneumoniae* | 1* | - | - | - | + | - | - | + | - | *S. pneumoniae* | 2.9 |
| Serotype 14 | Sanger serotype 14 | *S. pneumoniae* | 1* | 1* | - | - | + | - | - | + | - | *S. pneumoniae* | 3.2 |
| Serotype 18C | Sanger serotype 18C | *S. pneumoniae* | 1* | - | - | - | + | - | - | + | - | *S. pneumoniae* | 2.7 |
| Serotype 19F | Sanger serotype 19F | *S. pneumoniae* | 1* | 2* | - | - | + | - | - | + | - | *S. pneumoniae* | 2.9 |
| Serotype 19A | Sanger serotype 19A | *S. pneumoniae* | 1* | 1* | - | - | + | - | - | + | - | *S. pneumoniae* | 2.7 |
| Serotype 23F | Sanger serotype 23F | *S. pneumoniae* | 1* | - | - | - | + | - | - | + | - | *S. pneumoniae* | 3.2 |
| Non-capsular | CDC/21 | *S. pneumoniae* | 1* | - | - | - | + | - | - | + | - | *S. pneumoniae* | 2.8 |
| Non-capsular | CDC/22 | *S. pneumoniae* | 1* | - | - | - | + | - | - | + | - | *S. pneumoniae* | 2.7 |
| Non-capsular | CDC/23 | *S. pneumoniae* | 1* | - | - | - | + | - | - | + | - | *S. pneumoniae* | 2.5 |
| Non-capsular | CDC/24 | *S. pneumoniae* | 1* | - | - | - | + | - | - | + | - | *S. pneumoniae* | 3 |
| Non-capsular | CDC/25 | *S. pneumoniae* | 1* | - | - | - | + | - | - | + | - | *S. pneumoniae* | 3 |
| Non-capsular | CDC/26 | *S. pneumoniae* | 1* | - | - | - | + | - | - | + | - | *S. pneumoniae* | 2.9 |
| Non-capsular | CDC/27 | *S. pseudopneumoniae* | 2* | - | - | - | + | - | - | + | - | *S. pneumoniae* | 2.9 |
| Non-capsular | CDC/28 | *S. pneumoniae* | 2* | 1* | - | - | + | - | - | + | - | *S. pneumoniae* | 3.2 |
| Non-capsular | CDC/29 | *S. pneumoniae* | 1* | - | - | - | + | - | - | + | - | *S. pneumoniae* | 3.2 |
| Non-capsular | CDC/30 | *S. pneumoniae* | 1* | - | - | - | + | - | - | + | - | *S. pneumoniae* | 2.7 |
| Non-capsular | CDC/31 | *S. pneumoniae* | 1* | - | - | - | + | - | - | + | - | *S. pneumoniae* | 2.9 |
| Non-capsular | CDC/32 | *S. pneumoniae* | 1* | - | - | - | + | - | - | + | - | *S. pneumoniae* | 3.1 |
| Non-capsular | CDC/33 | *S. pneumoniae* | 1* | - | - | - | + | - | - | + | - | *S. pneumoniae* | 2.8 |
| Non-capsular | CDC/34 | *S. pneumoniae* | 1* | - | - | - | + | - | - | + | - | *S. pneumoniae* | 3.3 |
| Non-capsular | CDC/35 | *S. pneumoniae* | 1* | - | - | - | + | - | - | + | - | *S. pneumoniae* | 3.2 |
| Non-capsular | Kilian/13725 | *S. pneumoniae* | 1* | - | - | - | + | - | - | + | - | *S. pneumoniae* | 2.2 - 3.3 |
| Non-capsular | Kilian/SK1932/14860 | *S. pneumoniae* | 1* | - | - | - | + | - | - | + | - | *S. pneumoniae* | 2.9 - 3.3 |
| Non-capsular | Kilian/SK1933/A39557 | *S. pneumoniae* | 1* | - | - | - | + | - | - | + | - | *S. pneumoniae* | 2.1 – 2.5 |
| Non-capsular | Kilian/SK1939/A8061 | *S. pneumoniae* | 1* | - | - | - | + | - | - | + | - | *S. pneumoniae* | 2.4 – 2.8 |
| Non-capsular | Kilian/SK1934/A12931 | *S. pneumoniae* | 1* | - | - | - | + | - | - | + | - | *S. pneumoniae* | 3.0 – 3.6 |
| Non-capsular | Kilian/SK1938/A9003 | *S. pneumoniae* | 1* | 1* | - | - | + | - | - | + | - | *S. pneumoniae* | 2.9 – 3.2 |
| Non-capsular | Kilian/SK1935/A7890 | *S. pneumoniae* | 1* | - | - | - | + | - | - | + | - | *S. pneumoniae* | 2.8 – 3.1 |
| Non-capsular | Kilian/SK1941/A2009 | *S. pneumoniae* | 1* | - | - | - | + | - | - | + | - | *S. pneumoniae* | 2.0 -2.5 |
| Non-capsular | Kilian/SK1936/A4708 | *S. pneumoniae* | 1* | - | - | - | + | - | - | + | - | *S. pneumoniae* | 2.8 – 3.4 |
| Non-capsular | Kilian/SK1940/A39363 | *S. pneumoniae* | 1* | - | - | - | + | - | - | + | - | *S. pneumoniae* | 2.4 - 2.7 |
| *S. pseudopneumoniae* | CCUG49455 | *S. pseudopneumoniae* | 1* | - | + | - | + | + | - | + | - | *S. pseudopneumoniae* | 0.2 - 1.9 |
| *S. pseudopneumoniae* | 276-03 | *S. pneumoniae* | 2* | - | + | - | + | + | - | + | - | *S. pseudopneumoniae* | 0.5 - 1.5 |
| *S. pseudopneumoniae* | 61-14 | *S. oralis* | 2* | 3* | + | - | + | + | - | + | - | *S. pseudopneumoniae* | 1.7 – 1.8 |
| *S. pseudopneumoniae* | 338-14 | *S. pneumoniae* | 2* | - | + | - | + | + | - | + | - | *S. pseudopneumoniae* | 1.6 – 2.0 |
| *S. pseudopneumoniae* | 565-14 | *S. pseudopneumoniae* | 3* | - | + | - | + | + | - | - | - | *S. pseudopneumoniae* | 1.8 |
| *S. pseudopneumoniae* | 175/11 | *S. pseudopneumoniae* | 2* | 3* | + | - | + | + | - | + | - | *S. pseudopneumoniae* | 1.8 |
| *S. pseudopneumoniae* | 141/10 | *S. pseudopneumoniae* | 1* | 4* | + | - | + | + | - | + | - | *S. pseudopneumoniae* | 1.8 – 2.2 |
| *S. pseudopneumoniae* | SK1516 | *S. pneumoniae* | 2 | 3* | + | - | + | + | - | + | - | *S. pseudopneumoniae* | 1.2 – 1.4 |
| *S. pseudopneumoniae* | SK674 | *S. pneumoniae* | 2* | 2 | + | - | + | + | - | + | - | *S. pseudopneumoniae* | 0.2 – 0.3 |
| *S. mitis* | 172/10 | *S. oralis* | 3* | - | - | - | - | - | - | - | + | *S. mitis/S. oralis* | 0.1 |
| *S. mitis* | 19-03 | *S. mitis* | 3* | - | - | - | + | - | - | + | + | *S. mitis* | 0.1 |
| *S. mitis* | 187-03 | *S. mitis* | 3* | - | - | - | + | - | - | + | + | *S. mitis* | 0.8 |
| *S. mitis* | 24-03 | *S. oralis* | 3* | - | - | - | + | - | - | + | + | *S. mitis* | 0.3 |
| *S. mitis* | 68-03 | *S. mitis* | 3* | - | - | - | + | - | - | + | + | *S. mitis* | 0.4 |
| *S. mitis* | 72-03 | *S. mitis* | 2* | - | - | - | + | - | - | + | + | *S. mitis* | 0 |
| *S. mitis* | 108-03 | *S. pseudopneumoniae* | 4* | - | - | - | - | - | - | - | + | *S. mitis/S. oralis* | 0 |
| *S. mitis*  (Autolysin gene) | 24-16 | *S. pneumoniae* | 3* | - | - | - | + | - | - | + | + | *S. mitis* | 2.4 – 2.7 |
| *S. mitis* | SK642 | *S. mitis* | 3* | 1 | - | - | + | - | - | + | + | *S. mitis* | 0 |
| *S. mitis* | SK637 | *S. mitis* | 3* | - | - | - | + | - | - | + | + | *S. mitis* | 0 – 0.2 |
| *S. mitis* | SK271 | *S. pneumoniae* | 1 | 3* | - | - | + | - | - | + | - | *S. pneumoniae* | 0.1 – 0.2 |
| *S. mitis* | SK142 | *S. oralis* | 3* | - | - | - | + | + | - | + | + | *S. mitis* | 0 |
| *S. mitis*  (Autolysin gene) | SK564 | *S. mitis* | 3* | - | - | - | + | - | - | + | + | *S. mitis* | 0.7 – 1.3 |
| *S. mitis* | SK137 | *S. pneumoniae* | 2* | 3* | - | - | + | - | - | + | - | *S. pneumoniae* | 0 – 0.2 |
| *S. mitis* | SK1126 | *S. oralis* | 1 | 2 | - | - | + | - | - | + | - | *S. pneumoniae* | 0 – 0.2 |
| *S. mitis*  (Autolysin gene) | SK597 | *S. oralis* | 1 | 3* | + | - | - | - | - | - | + | *S. mitis* | 1.1 – 1.4 |
| *S. mitis* | SK608 | *S. mitis* | 3* | - | - | - | + | - | - | - | + | *S. mitis* | 0.1 – 0.3 |
| *S. mitis* | SK321 | *S. mitis* | 3* | - | + | - | + | - | - | + | + | *S. mitis* | 0 |
| *S. mitis* | SK113 | *S. oralis* | 1 | 1 | - | + | - | - | + | - | + | *S. mitis/ S. oralis* | 0 – 0.3 |
| *S. mitis* | SK578 | *S. mitis* | 1* | 3* | - | - | + | - | - | + | + | *S. mitis* | 0 |
| *Streptococcus oralis* | 10-03 | *S. oralis* | 1* | 2* | - | + | - | - | + | - | - | *S. oralis* | 0 |
| *Streptococcus australis* | 20-03 | *S. parasanguinis* | - | 3* | - | - | - | - | - | - | - | ? | 0.1 |
| *Streptococcus oralis* | 18-03 | *S. oralis* | 1* | - | - | + | - | - | + | - | + | *S. mitis/S. oralis* | 0.1 |
| *Streptococcus australis* | 25-03 | *S. parasanguinis* | 1 | 2* | - | - | - | - | - | - | - | ? | 0.1 |
| *S. sanguinis* | 49-03 | *S. sanguinis* | 1* | 1* | - | - | - | - | - | - | + | ? | 0 |
| *S. sanguinis* | 122-03 | *S. sanguinis* | 1* | 1* | - | - | - | - | - | - | + | ? | 0.3 |
| *S. sanguinis* | 185-03 | *S. sanguinis* | 1* | 1* | - | - | - | + | - | - | + | ? | 0.3 |
| *S. sanguinis* | 222-03 | *S. sanguinis* | 1* | 1* | - | - | - | + | - | - | - | ? | 0 |

Supplementary table 2. Repeated testing of 25 strains over time and performed by different persons. OD-values below 0 are considered as 0.0.

| *Defined species* | Strains identification | Bile test  (03-03-2016)  (Person 1) | Bile test  (09-03-2016)  (Person 1) | Bile test  (17-03-2016)  (Person 2) | Bile test  (17-03-2016)  (Person 1) | Bile test  (23-08-2016)  (Person 1) | Bile test  (23-08-2016)  (Person 3) | Total range of test (OD-values) |
| --- | --- | --- | --- | --- | --- | --- | --- | --- |
| *S. pneumoniae* | ATCC49619 | 3 | 3.4 | 2.9 | 3.4 | 3.3 | 3.2 | 2.9 – 3.5 (0.6) |
| *S. pneumoniae* Non-capsular | Kilian/13725 | 3.1 | 3.3 | Not done | Not done | 2.2 | 2.6 | 2.2 – 3.3 (1.1) |
| *S. pneumoniae* Non-capsular | Kilian/14860 | 3.2 | 3.3 | Not done | Not done | 2.9 | 3 | 2.9 – 3.3 (0.4) |
| *S. pneumoniae* Non-capsular | Kilian/A39557 | 2.5 | 2.4 | Not done | Not done | 2.1 | 2.2 | 2.1 – 2.5 (0.4) |
| *S. pneumoniae* Non-capsular | Kilian/A8061 | 2.6 | 2.8 | Not done | Not done | 2.4 | 2.6 | 2.4 – 2.8 (0.4) |
| *S. pneumoniae* Non-capsular | Kilian/A12931 | 3.1 | 3.6 | Not done | Not done | 3 | 3.2 | 3 – 3.6 (0.6) |
| *S. pneumoniae* Non-capsular | Kilian/A9003 | 3 | 3.2 | Not done | Not done | 2.9 | 3 | 2.9 – 3.2 (0.3) |
| *S. pneumoniae* Non-capsular | Kilian/A7890 | 3.1 | 2.9 | Not done | Not done | 2.8 | 2.9 | 2.8 – 3.1 (0.3) |
| *S. pneumoniae* Non-capsular | Kilian/A2009 | 2.1 | 2.5 | Not done | Not done | 2 | 2.3 | 2 – 2.5 (0.5) |
| *S. pneumoniae* Non-capsular | Kilian/A4708 | 3.2 | 2.9 | Not done | Not done | 2.8 | 3.4 | 2.8 – 3.4 (0.6) |
| *S. pneumoniae* Non-capsular | Kilian/A39363 | Not done | 2.9 | Not done | Not done | 2.4 | 2.8 | 2.4 – 2.9 (0.5) |
| *S. pseudopneumoniae* | Kilian/SK1516 | 1.2 | 1.4 | Not done | Not done | 1.4 | 1.4 | 1.2 – 1.4 (0.2) |
| *S. pseudopneumoniae* | Kilian/SK674 | Not done | Not done | 0.3 | 0.2 | 0 | 0.4 | 0 – 0.4 (0.4) |
| *S. mitis* | Kilian/SK642 | Not done | Not done | 0 | 0 | 0 | 0 | 0 - 0 (0) |
| *S. mitis* | Kilian/SK637 | Not done | Not done | 0 | 0.2 | 0 | 1.2 | 0 – 1.2 (1.2) |
| *S. mitis* | Kilian/SK271 | Not done | Not done | 0.2 | 0.1 | 0 | 0 | 0 – 0.2 (0.2) |
| *S. mitis* | Kilian/SK142 | Not done | Not done | 0 | 0 | 0.2 | 0 | 0 – 0.2 (0.2) |
| *S. mitis* | Kilian/SK564 | Not done | Not done | 0.7 | 1.3 | 0.4 | 1.3 | 0.4-1.3 (0.9) |
| *S. mitis* | Kilian/SK137 | Not done | Not done | 0.2 | 0 | 0 | 0 | 0 - 0.2 (0.2) |
| *S. mitis* | Kilian/SK1126 | Not done | Not done | 0 | 0.2 | 0 | 0 | 0 – 0.2 (0.2) |
| *S. mitis* | Kilian/SK597 | Not done | Not done | 1.1 | 1.4 | 0.3 | 0.2 | 0.2 – 1.4 (1.2) |
| *S. mitis* | Kilian/SK608 | Not done | Not done | 0.3 | 0.1 | 0 | 0 | 0 – 0.3 (0.3) |
| *S. mitis* | Kilian/SK321 | Not done | Not done | 0 | 0 | 0 | 0 | 0 – 0 (0) |
| *S. mitis* | Kilian/SK113 | Not done | Not done | 0.3 | 0 | 0 | 0.1 | 0 – 0.3 (0.3) |
| *S. mitis* | Kilian/SK578 | Not done | Not done | Not possible | Not possible | 0 | 0 | 0 – 0 (0) |
